# Automated patch clamp data improve variant classification and penetrance stratification for *SCN5A*–Brugada syndrome

**DOI:** 10.1093/eurheartj/ehaf874

**Published:** 2025-11-18

**Authors:** Matthew J O’Neill, Joanne G Ma, Jessa L Aldridge, Joseph F Solus, Genevieve R Harvey, Paige H Roberson, Julien Barc, Connie R Bezzina, Dan M Roden, Roddy Walsh, Jamie I Vandenberg, Andrew M Glazer, Chai-Ann Ng

**Affiliations:** Department of Medicine, Brigham and Women’s Hospital, Boston, MA, USA; Clinical Fellow in Medicine, Harvard Medical School, Boston, MA, USA; Mark Cowley Lidwill Research Program in Cardiac Electrophysiology, Victor Chang Cardiac Research Institute, 405 Liverpool St, Darlinghurst, NSW, Australia; School of Clinical Medicine, St Vincent’s Healthcare Clinical Campus, Faculty of Medicine and Health, University of New South Wales, Sydney, Australia; Vanderbilt Center for Arrhythmia Research and Therapeutics (VanCART), Division of Clinical Pharmacology, Department of Medicine, Vanderbilt University Medical Center, 2215B Garland Ave, 1235 Medical Research Building IV, Nashville, TN, USA; Vanderbilt Center for Arrhythmia Research and Therapeutics (VanCART), Division of Clinical Pharmacology, Department of Medicine, Vanderbilt University Medical Center, 2215B Garland Ave, 1235 Medical Research Building IV, Nashville, TN, USA; Vanderbilt Center for Arrhythmia Research and Therapeutics (VanCART), Division of Clinical Pharmacology, Department of Medicine, Vanderbilt University Medical Center, 2215B Garland Ave, 1235 Medical Research Building IV, Nashville, TN, USA; Vanderbilt Center for Arrhythmia Research and Therapeutics (VanCART), Division of Clinical Pharmacology, Department of Medicine, Vanderbilt University Medical Center, 2215B Garland Ave, 1235 Medical Research Building IV, Nashville, TN, USA; Nantes Université, CNRS, INSERM, l’institut du thorax, IRS-UN, 8 Quai Moncousu, BP 70721, Nantes 44007, France; European Reference Network for Rare, Low Prevalence and Complex Diseases of the Heart: ERN GUARD-Heart, Amsterdam, The Netherlands; European Reference Network for Rare, Low Prevalence and Complex Diseases of the Heart: ERN GUARD-Heart, Amsterdam, The Netherlands; Department of Experimental Cardiology, Amsterdam Cardiovascular Sciences, University of Amsterdam, Amsterdam UMC, Amsterdam, The Netherlands; Vanderbilt Center for Arrhythmia Research and Therapeutics (VanCART), Division of Clinical Pharmacology, Department of Medicine, Vanderbilt University Medical Center, 2215B Garland Ave, 1235 Medical Research Building IV, Nashville, TN, USA; Department of Pharmacology, Vanderbilt University Medical Center, Nashville, TN, USA; Department of Biomedical Informatics, Vanderbilt University Medical Center, Nashville, TN, USA; Department of Experimental Cardiology, Amsterdam Cardiovascular Sciences, University of Amsterdam, Amsterdam UMC, Amsterdam, The Netherlands; Cardiovascular and Genomics Research Institute, City St. George’s University of London, London, UK; Mark Cowley Lidwill Research Program in Cardiac Electrophysiology, Victor Chang Cardiac Research Institute, 405 Liverpool St, Darlinghurst, NSW, Australia; School of Clinical Medicine, St Vincent’s Healthcare Clinical Campus, Faculty of Medicine and Health, University of New South Wales, Sydney, Australia; Vanderbilt Center for Arrhythmia Research and Therapeutics (VanCART), Division of Clinical Pharmacology, Department of Medicine, Vanderbilt University Medical Center, 2215B Garland Ave, 1235 Medical Research Building IV, Nashville, TN, USA; Mark Cowley Lidwill Research Program in Cardiac Electrophysiology, Victor Chang Cardiac Research Institute, 405 Liverpool St, Darlinghurst, NSW, Australia; School of Clinical Medicine, St Vincent’s Healthcare Clinical Campus, Faculty of Medicine and Health, University of New South Wales, Sydney, Australia

**Keywords:** Arrhythmia, Brugada syndrome, Automated patch clamp, Variant classification, Computational prediction

## Abstract

**Background and Aims:**

Brugada Syndrome (BrS) is an inherited arrhythmia disorder that causes an elevated risk of sudden cardiac death. Approximately 20% of patients with BrS have rare variants in *SCN5A*, which encodes the cardiac sodium channel Na_V_1.5. Genetic workup of BrS is often complicated by *SCN5A* variants of uncertain significance (VUS) and/or incomplete penetrance. This study deployed an *SCN5A*-BrS functional assay at cohort scale to facilitate the implementation of genetic and precision medicine.

**Methods:**

All 252 missense and in-frame insertion/deletion *SCN5A* variants from a previously published large cohort of BrS cases (*n* = 3335 patients) were analysed using a calibrated high-throughput automated patch-clamp (APC) assay. Variant functional *Z*-scores were assigned evidence levels ranging from BS3_moderate (normal function) to PS3_strong (loss-of-function), as defined by American College of Medical Genetics and Genomics criteria. Functional evidence was combined with population frequency, hotspot, case counts, protein-length changes, and in silico predictions. Odds ratios of BrS case–control enrichment and penetrance for BrS were calculated from variant frequencies in the BrS cohort and in gnomAD.

**Results:**

Most variants (146/252) were functionally abnormal (*Z* ≤ −2), with 100 having severe loss-of-function (*Z* ≤ −4). Functional evidence enabled the reclassification of 110 of 225 VUS; 104 to likely pathogenic and 6 to likely benign. *SCN5A* variants with loss-of-function were mainly localized to the transmembrane domains, especially the regions comprising the central pore. *SCN5A* variant penetrance was proportional to the severity of loss-of-function; variants with Z ≤ −6 had penetrance of 24.5% (15.9%–37.7% CI) and an odds ratio of 501 for BrS.

**Conclusions:**

This cohort-scale APC dataset stratifies *SCN5A* variants found in BrS patients into normal function ‘bystander’ variants that have a low risk of BrS and loss-of-function variants that have a high risk for BrS. Functional data can be integrated with other criteria to reclassify a substantial fraction of VUS. The dataset helps clarify the *SCN5A*–BrS relationship and will improve the diagnosis and clinical management of BrS probands and their families.

## Structured Graphical abstract

**Structured Graphical abstract:**
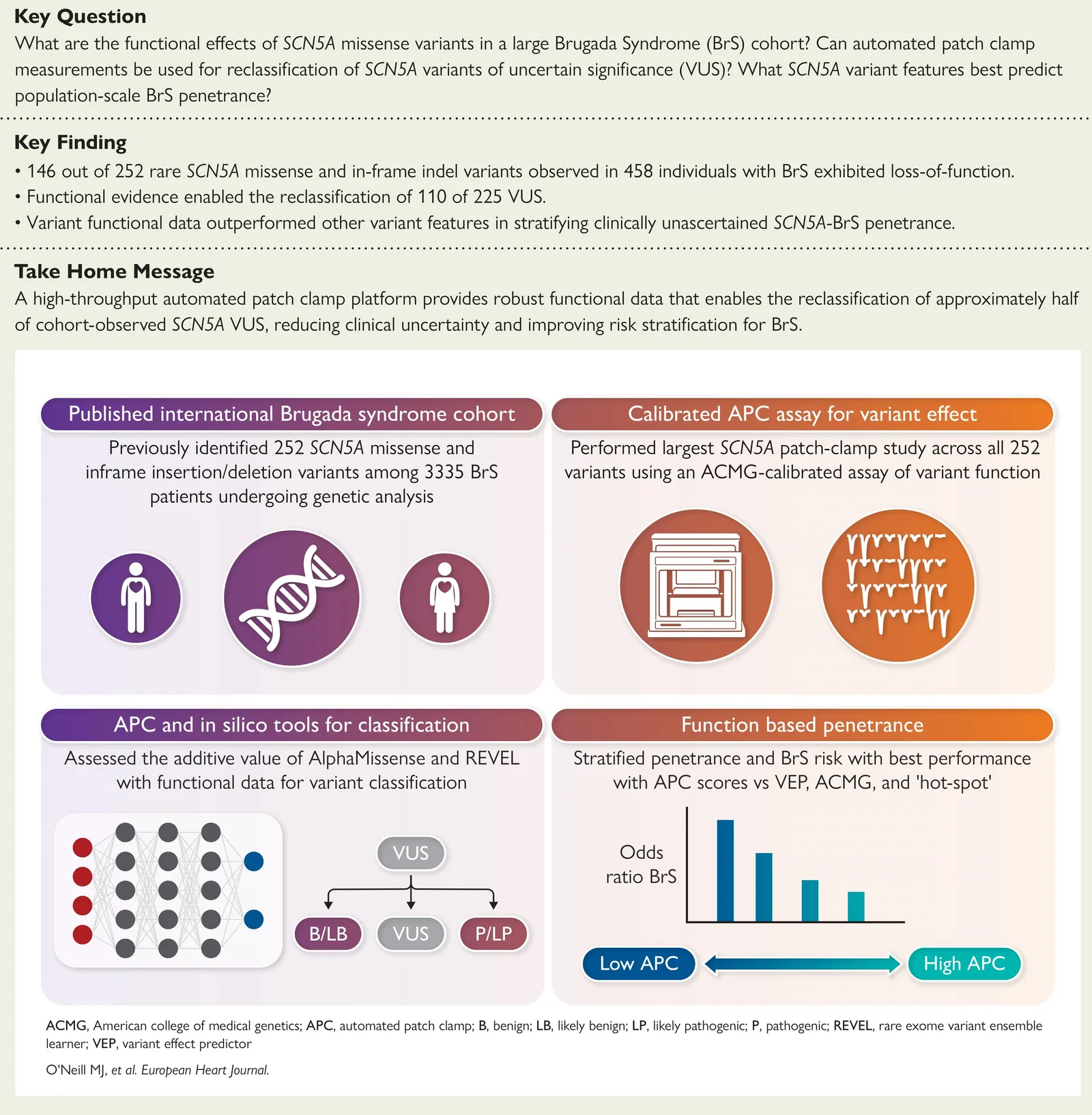

Integrated functional and computational assessment of *SCN5A* variants in Brugada syndrome. We assayed 252 *SCN5A* missense and in-frame insertion/deletion variants, from an international BrS cohort, using an ACMG-calibrated automatic patch-clamp (APC) assay–the largest *SCN5A* functional dataset to date. Combining APC scores with *in silico* predictors (AlphaMissense, REVEL) improved ACMG-based variant classification and reclassification of VUS. Functional data further stratified penetrance and BrS risk, outperforming computational predictors, ACMG criteria, and positional “hot-spot“ models.

**See the editorial comment for this article ‘Reducing the variant of uncertain significance burden in inherited cardiac disorders: from variant to function to action?’, by C.A. Remme and J. van der Velden, https://doi.org/10.1093/eurheartj/ehaf1032.**

Translational perspective *SCN5A* is the only gene definitively linked to Brugada syndrome (BrS). Approximately 20% of patients carry *SCN5A* variants, yet many are classified as variants of uncertain significance (VUS), which complicates care. This study reports the first cohort-scale, high-precision automated patch-clamp study that enables VUS reclassification and estimates variant penetrance when *SCN5A* findings emerge as secondary findings in unselected populations.

## Introduction

Brugada Syndrome (BrS; MIM:601144) is a heritable arrhythmia syndrome characterized by ST-segment elevation in the right precordial leads on the electrocardiogram, increased risk for severe ventricular arrhythmias, and sudden death.^1^ Early diagnosis of BrS is critical, as arrhythmias or sudden cardiac death can be the first manifestation of the disease,^2,3^ and treatments such as medication, ablation, or an implanted cardioverter defibrillator can be lifesaving.^4^

Large-effect size variants in over 20 genes have been suggested to cause BrS. However, an international consensus group found that rare variants in only *SCN5A* have definitive evidence for causing BrS.^5^  *SCN5A* encodes the cardiac voltage-gated sodium channel Na_V_1.5, which is responsible for depolarization at the start of the cardiac action potential.^6^ Rare loss-of-function (LOF) variants in *SCN5A* are present in 15%–30% of BrS cases, depending on ancestry and disease sensitivity.^7^ A high common genetic variant burden can also contribute to BrS risk or modify the penetrance of rare *SCN5A* variants.^1,8,9^ Notably, BrS has a large degree of incomplete penetrance and only a fraction of carriers with the same rare genetic variant present with BrS.^8^

The American College of Medical Genetics and Genomics/Association for Molecular Pathology (ACMG/AMP) has established criteria for variant classification in medically important genes, including *SCN5A*.^10,11^ These criteria include allele frequency (AF), co-segregation with disease, location in protein hot-spots, *in silico* prediction tools, and laboratory functional experiments.^10^ Variant classifications range from Benign/Likely Benign (B/LB) to Pathogenic/Likely Pathogenic (P/LP). However, as most missense variants in *SCN5A* are only observed in a small number of individuals, there is often insufficient evidence to permit definitive classification, and they are instead labelled Variants of Uncertain Significance (VUS). To help overcome the burden of VUS, an international consortium of geneticists, genomic scientists, and clinicians (Clinical Genome Resource, ClinGen) has made recommendations for implementation of the ACMG/AMP variant classification standards with a greater emphasis on validation of prediction algorithms^12^ and functional assays^13^ using sufficient variant controls.

Until recently, functional data have been sparsely available for inherited arrhythmia syndromes, where traditional manual patch-clamp studies of ion channel variants are time-intensive and low-throughput. To overcome this limitation, we developed a high-throughput automated patch-clamp (APC) assay to investigate *SCN5A* variant function.^14^ The assay has been calibrated on a large set of 25 B/LB and 24 P/LP variants, showing excellent discrimination between these two sets of variants. The assay uses a protocol to interrogate functions under conditions that mimic physiological context, i.e. using a −90 mV holding potential, which is close to the resting potential in native cardiomyocytes, so that variants that affect the voltage dependence of inactivation or activation, as well as variants that affect trafficking, will impact the magnitude of peak current density. The assay achieved a 95.8% sensitivity and 96% specificity for the classification of BrS variants and met ClinGen criteria for applying strong evidence for loss-of-function (PS3). Although the original data supported BS3_strong for normal function, we conservatively limited this to BS3_moderate.^13^

A recent publication of the genetic characteristics of 3335 BrS patients^9,15^ provides a valuable resource to explore the additive value of calibrated functional data^14^ to reclassify *SCN5A* VUS. The cohort includes 614 individuals with rare heterozygous *SCN5A* variants, spanning 353 unique variants: 103 predicted LOF variants (e.g. nonsense, frameshift, or splice-site variants) and 252 missense or in-frame insertion/deletion (indel) variants.^15^ Here, we provide calibrated APC data as *Z-*scores for the entire set of 252 rare *SCN5A* missense and in-frame indel variants previously identified in the published cohort of BrS patients.^15^ We then determine the value of adding this functional evidence, in conjunction with population frequency, hotspot, case enrichment, protein length, and *in silico* tools, to reclassify *SCN5A*-BrS cohort variants. Lastly, we calculate estimates of the relationship between variant-specific function and both case–control enrichment and BrS penetrance.

## Methods

### Variant cohort

We studied 252 rare missense and in-frame indel *SCN5A* variants harboured by 458 of 3335 individuals undergoing evaluation for BrS, as described by Walsh *et al*.^15^ Comprehensive variant information, including position, nucleotide, and amino acid changes, is described in Supplementary data online, *Table S1*. All experimental studies used the most common *SCN5A* transcript in the adult heart, which includes the adult isoform of exon 6 and a deletion of the alternatively spliced p.Gln1077 residue (ENST00000423572; MANE Select transcript).^16^ Our analysis uses the nomenclature corresponding to this 2015 residue isoform. However, we also present a second variant nomenclature corresponding to the full 2016 residue isoform that has often been used in the literature (ENST00000333535; Supplementary data online, *Table S1*). The missense variant p.Asn1379Lys was reported in two individuals via different nucleotide substitutions—c.4137C > G and c.4137C > A. Only c.4137C > A was studied (see Supplementary data online, *Table S1*).

### High-throughput automated patch-clamp methods

**Figure 1:**
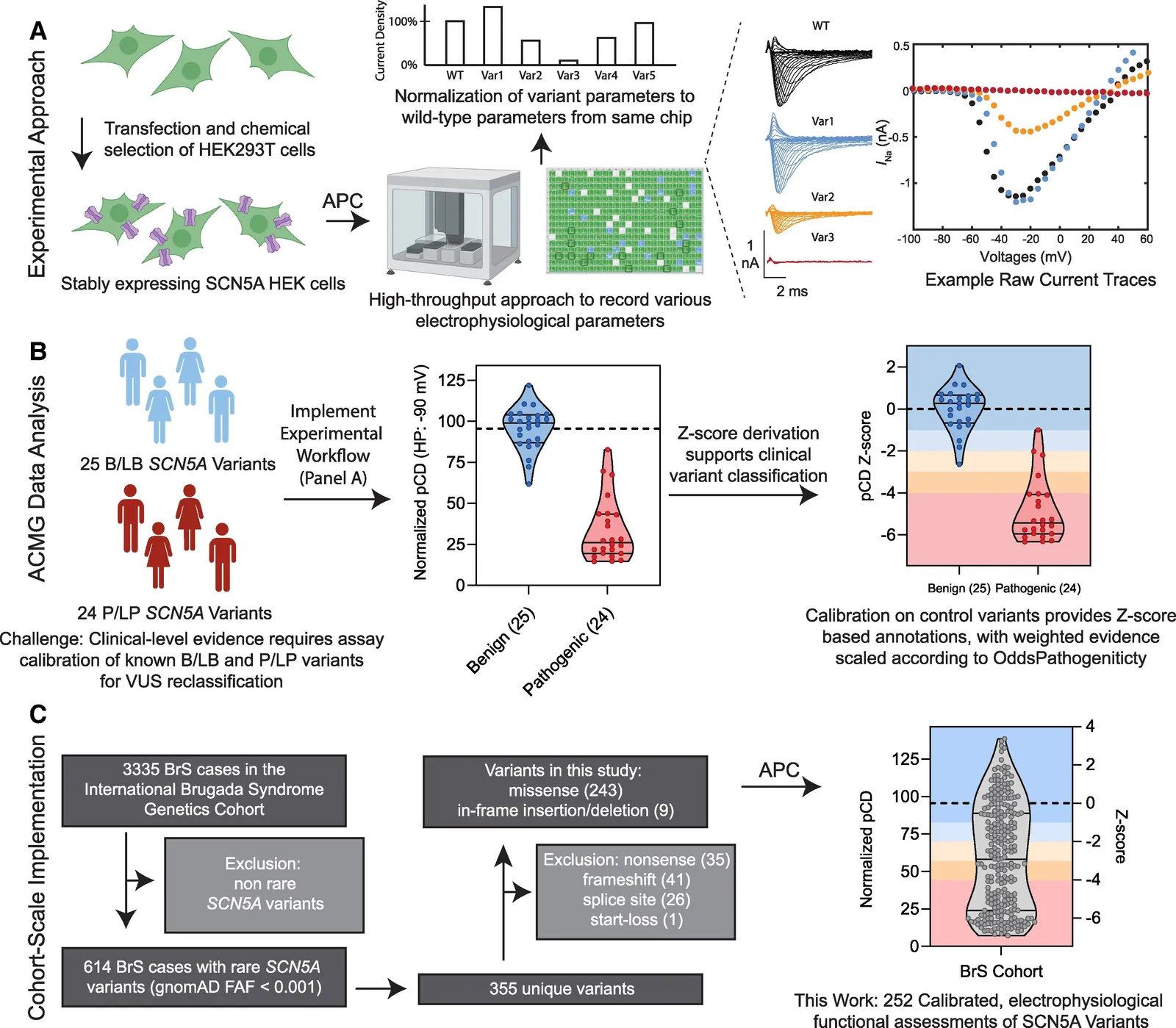
Schematic of project background and experimental design. *(A*) The automated patch-clamp (APC) assay measures electrophysiological properties of *SCN5A* variants that are stably expressed in HEK293 cell lines. Variant peak current densities are normalized to WT measurements made on the same plate. *(B*) For implementation into the ACMG/AMP framework, we report variant function as a *Z*-score, which is calculated as multiples of the standard deviation from the mean of 25 B/LB control variants. Datapoints indicate the median of each benign and pathogenic variant control, and the lines represent the mean and interquartile range of each group. *(C*) Here, we studied 252 missense and in-frame insertion/deletion variants observed among 458 patients undergoing evaluation for BrS. The APC uncovered a spectrum of effects among the studied channels. Datapoints indicate the median of each cohort variant (grey), and lines represent the mean and interquartile range of the cohort

*SCN5A* variants were experimentally studied at Vanderbilt University Medical Center (VUMC) and/or Victor Chang Cardiac Research Institute (VCCRI).^14^ Each site performed independent plasmid cloning, cell culture and transfections, APC assays, and data analysis (*Figure 1*). The VUMC group used manually cloned plasmids expressed stably in HEK-293T ‘landing-pad’ cell lines.^17^ The VCCRI group stably co-transfected commercially constructed *SCN5A* variant plasmids (Genscript Inc; Piscataway, NJ, USA) with the POG44 ‘flp-in’ vector into HEK293 cell lines.^18^ After selection for cells containing a single stable integration event, expression of Na_V_1.5 was induced by adding 250 μg/mL or 0.2 μg/mL doxycycline at VUMC or VCCRI, respectively. Cells were studied at each site using the SyncroPatch 384PE system (Nanion Technologies, Munich). The VUMC site used the following solutions: Internal solution (mM): 10 NaCl, 10 CsCl, 110 CsF, 10 HEPES, 10 EGTA; pH 7.2 with CsOH. External solution (mM): 80 NaCl, 60 N-methyl-D-glucamine, 1 MgCl_2_, 4 KCl, 2 CaCl_2_, 10 HEPES, 5 Glucose; pH 7.4 with HCl. The VCCRI used Internal solution (mM): 10 NaCl, 10 CsCl, 110 CsF, 10 HEPES, 10 EGTA; pH 7.2 with CsOH. External solution (mM): 20 NaCl, 5 KCl, 1 Mg, 2 Ca, 120 tetraethylammonium chloride, 10 HEPES, 5 Glucose; pH 7.4 with NaOH.

The peak sodium current was measured during a depolarizing step to −30 mV from a holding potential of −90 mV. After the application of quality control measures (see Supplementary data online, *Table S2*), we calculated the peak current density (pCD; pA/pF) by normalizing the peak current amplitude (pA) by cell capacitance (pF). The voltage dependence of activation was obtained by measuring the peak current amplitude in cells depolarized from a holding potential of −120 mV to voltages between −100 and 0 mV, at +5 mV increments. The voltage dependence of inactivation was obtained by holding each cell at voltages between −140 and −40 mV, at 5 mV increments, for 500 ms, then measuring the tail current amplitude at −30 mV. Steady state activation (SSA) and steady state inactivation (SSI) curves were fitted with a Boltzmann equation to determine the voltages for half activation and half inactivation (SSA *V*_50_ and SSI *V*_50_, respectively). Recovery from inactivation was determined by the relative peak current amplitude (current at test-pulse normalized to current at pre-pulse) with increasing time intervals between the two pulses. These data were fitted with a double exponential equation to calculate the time of half recovery (*T*_50_). Analysis of experimental data was performed in R or MATLAB (see Data and Code Availability below).

### Assay design using benign variant controls

The primary analysis used in this study involves the evaluation of pCD for variants measured after holding the membrane potential at −90 mV, to mimic the physiologically-relevant cardiomyocyte resting membrane potential, as described by Ma *et al*.^14^ This ‘physiological’ (−90 mV) assay was updated and expanded in the present study to include data recorded for all variant controls at both VUMC and VCCRI. The 25 benign controls were previously curated based on having an allele frequency in gnomAD v3.2 greater than the prevalence expected for BrS (filtering allele frequency, FAF ≥0.0003).^14^

Measurements of ‘physiological’ pCD show a non-Gaussian distribution, which can be converted to a Gaussian distribution using a square-root transformation.^14,19^ As high-throughput experiments profile large numbers of cells for each variant, the mean value for any given variant may be statistically significantly different from wild-type (WT) but not be of biological significance. To address this, we examined the variability of the 25 benign controls. Specifically, for each benign variant control, we determined a mean value by normalizing to WT controls recorded on the same plate. The mean data values (across cellular replicates) of these 25 benign controls were normally distributed, as evidenced by a QQ plot and Shapiro-Wilk normality test (*P* = .07, Supplementary data online, *Figure S1*). Since the distribution was normal, we used the mean and standard deviation of the benign controls to calculate *Z*-scores as the primary readout for interpreting biologically significant variant function.

### *Z*-score calculation and interpretation

The *Z*-score defines the number of standard deviations (SD) a variant's mean pCD differs from the mean of the 25 benign variants:


(1) $$Z_{\mathrm{variant}}=\frac{\mathrm{Mean}\;\mathrm{of}\;\mathrm{pC}D_{\mathrm{variant}}\;-\;\mathrm{Mean}\;\mathrm{of}\;\mathrm{pC}D_{\mathrm{benign}\;\mathrm{controls}}}{\mathrm{SD}\;\mathrm{of}\;\mathrm{pC}D_{\mathrm{benign}\;\mathrm{controls}}}$$

where *Z*_variant_ is the *Z*-score for each variant tested. Mean of pCD_variant_ refers to the average value of transformed current density for a variant, Mean of pCD_benign controls_ refers to the average value of transformed current density for all 25 benign controls, SD of pCD_benign controls_ refers to the standard deviation of transformed current density for all 25 benign controls.

In this study, the terms ‘normal function’, ‘abnormal function’, and ‘LOF’ refer to variant function as measured by patch clamp in a hemizygous HEK293 cell model. Normal function is defined by *Z* > −2. Abnormal function (i.e. LOF) was categorized according to the severity of LOF: mild LOF (−2 ≥ *Z* > −3), moderate LOF (−3 ≥ *Z* > −4) or severe LOF (*Z* ≤ −4). It is important to note that this classification applies only to *SCN5A*-related BrS, and these experimental results may not accurately describe *SCN5A* variant effects for other clinical phenotypes.

To harmonize the VCCRI and VUMC datasets, we calculated a weighted average for each variant, weighting each site's measurements by the number of cells recorded:


(2) $$Weighted\;average=VCCRI\;result*\left(\frac{\#\;cells\;VCCRI}{total\;\#\;cells}\right)+VUMC\;result*\left(\frac{\#\;cells\;VUMC}{total\;\#\;cells}\right)$$

where VCCRI result and VUMC result are the mean values obtained at each laboratory, # cells VCCRI and # cells VUMC are the number of cells recorded at each laboratory, and total # cells is the total number of cells across the two research laboratories.

We also performed a secondary analysis based on combined pCD and gating parameters. Inclusion of gating parameters, however, did not improve variant classifications nor correlation with BrS penetrance.

### Prediction of variant splicing impacts

Comprehensive variant effects may be obscured in our cDNA-based APC assay, which cannot account for variant splicing effects, given that cDNA does not contain introns. To consider potential variant effects on splicing, we obtained SpliceAI scores from the SpliceAI website (https://spliceailookup.broadinstitute.org/), using default parameters (hg38, Gencode basic, max distance 500 bp, no masked scores).^20^ This algorithm generates scores for each single-nucleotide variant that correspond to the predicted molecular events of native splice acceptor and donor loss or gain. To collapse these four values into a single score, we calculated aggregate SpliceAI scores using the formula:


(3) P(aberrantsplicing)=1–((1–AG)*(1–AL)*(1–DG)*(1–DL))

where P (aberrant splicing) is the aggregate probability of aberrant splicing, and AG, AL, DG, and DL indicate scores for Acceptor Gain, Acceptor Loss, Donor Gain, and Donor Loss, respectively. This aggregate score has good sensitivity and specificity for detecting *SCN5A* variant splicing effects.^21^ Because the primary focus of this paper is to determine the predictive ability of the APC assay, we present the SpliceAI scores in Table S3, but did not integrate them towards variant classification.

### Cohort-level case–control enrichment analysis

Analyses of allele frequency used the gnomAD v4.1 FAF metric. FAF corresponds to the maximum allele frequency across major continental ancestries, with additional statistical adjustments.^22^ We defined rare variants as 0.001 > FAF >0.00001 and ultra-rare variants as FAF ≤0.00001. The proportion of European-ancestry BrS cases (*n* = 2400) carrying rare variants was calculated for the entire Na_V_1.5 protein and for different topological regions. The topological regions were defined by visual inspection of the cryo-EM structures 6QLA.pdb^23^ and 8VYJ.pdb^24^ in Chimera, using the S4/S5 linker and the top of the transmembrane helices as the boundaries for the lipid membrane. We define the following regions: a) Pore loop regions I–IV: residues 273–389, 862–912, 1356–1444, 1679–1743; b) Transmembrane regions (non-pore loops) I–IV: residues 132–410, 718–938, 1206–1469, 1528–1771 (excluding pore loop region residues); c) N-terminus: residues 1–131; d) Interdomain linker (IDL) regions: residues 411–717, 939–1205, 1470–1527; and e) C-terminus: residues 1772–2015. We also cross-checked these ranges using the Positioning of Proteins in Membranes (PPM) server^25^ and obtained highly similar transmembrane ranges. For each region, the proportion of cases carrying ultra-rare or rare variants was sub-classified according to the ACMG/AMP functional (PS3/BS3) evidence applied or the overall ACMG/AMP classification. Control frequencies were calculated using the non-Finnish European samples of the gnomAD v3 genomes dataset, as it has a better breadth of coverage with whole genome sequencing compared to whole exome sequencing for calculating overall control frequencies.

### ACMG/AMP criteria implementation and evaluation

Variant functional data (PS3/BS3) were assigned using our *Z*-score approach.^14,26^ Variants were binned by *Z*-score according to degree of LOF, ranging from PS3 to BS3_moderate: PS3_strong: *Z* ≤ −4; PS3_mod −4 < *Z* ≤ −3; PS3_supp −3 < *Z* ≤ −2; BS3_supp −2 < *Z* ≤ −1; BS3_mod *Z* > −1. For variant AF, we used population data from gnomAD v4.1,^22^ implementing PM2_supporting (FAF <0.00003) and BS1 (max population AF >0.0003).^14^ Case enrichment (PS4) for single variants was implemented as described by Walsh *et al*.^15^ In-frame indel variants received PM4 evidence due to a change in protein length. The hotspot criterion (PM1) was assessed based on location within the protein and gnomAD AF stratified by genetic ancestry (European vs East Asian; see differential *SCN5A*-BrS burden in Walsh *et al*)^15^ to provide up to moderate-level evidence. Two *in silico* predictors were used to apply computational variant effect criteria (PP3/BP4): REVEL^27^ and AlphaMissense.^28^ We present sensitivity analyses of each variant effect predictor by limiting the combined PP3/PM1 criteria to strong, based on a previous calibration by ClinGen Sequence Variant Interpretation Working Group.^29^ Thresholds for each criterion implementation are outlined in *Table 1*. Each criterion strength was assigned a point value according to the ACMG Bayesian points-based system (*Table 2A*).^30^ Criteria were combined by adding or subtracting points for eventual classification. Under the Bayesian point scale system,^30^ final point scores ≥10 or ≥6 were required for pathogenic classifications P or LP, respectively, and final point scores ≤−1 or ≤−7 were required for benign classifications LB or B, respectively. However, to classify a variant as LB, we used a more conservative cut-off of −5 points, which corresponds to 1 strong benign and 1 supporting benign criteria in the 2015 ACMG/AMP guidelines (*Table 2B*, Supplementary data online, *Table S4*).^10^

**Table 1:** ACMG/AMP evidence criteria applied in this study

Evidence class	Rule	Criteria applied
Frequency	PM2_supporting	gnomADv4 exomes AF <0.00003
	BS1/BA1	gnomADv4 exomes FAF ≥0.0003
Case enrichment (single variant)	PS4_strong	≥20
	PS3_moderate	≥10 & <20
	PS4_supporting	≥5 & <10
Hotspot enrichment	PM1_moderate	Transmembrane region, if gnomADv4 FAF <0.00001 and detected in a European-ancestry case
	PM1_supporting	Transmembrane region, if gnomADv4 FAF <0.00001 and detected only in a Japanese ancestry case
	PM1_supporting	N-Terminus region, if gnomADv4 FAF <0.00001 and detected in a European-ancestry case
	PM1_supporting	C-Terminus region, if gnomADv4 FAF <0.00001 and detected in case of either ancestry
Functional studies	BS3_moderate	−1 < *Z* < 1
	BS3_supporting	−2 < *Z* < −1
	PS3_supporting	−3 < *Z* < −2
	PS3_moderate	−4 < *Z* < −3
	PS3_strong	*Z* ≤ −4
**Computational evidence approaches**		
AlphaMissense_MaxSupporting	PP3_supporting	Any AlphaMissense score ≥0.792—BUT PP3 not exceeding SUPPORTING
	BP4_supporting	AlphaMissense score ≤0.169
AlphaMissense_MaxStrong (total PP3/PM1 not exceeding STRONG)	PP3_strong	AlphaMissense score ≥0.990—BUT total PP3/PM1 not exceeding STRONG
	PP3_+3	AlphaMissense score <0.989 & >0.972—BUT total PP3/PM1 not exceeding STRONG
	PP3_moderate	AlphaMissense score <0.971 & >0.906—BUT total PP3/PM1 not exceeding STRONG
	PP3_supporting	AlphaMissense score <0.905 & >0.792—BUT total PP3/PM1 not exceeding STRONG
	BP4_supporting	AlphaMissense score <0.169 & >0.100—BUT total PP3/PM1 not exceeding STRONG
	BP4_moderate	AlphaMissense score <0.099 & >0.071—BUT total PP3/PM1 not exceeding STRONG
	PP3_-3	AlphaMissense score ≤0.070
REVEL_MaxSupporting	PP3_supporting	Any REVEL score ≥0.644—BUT PP3 not exceeding SUPPORTING
	BP4_supporting	REVEL score ≤0.29
REVEL_MaxStrong (total PP3/PM1 not exceeding STRONG)	PP3_strong	REVEL score ≥0.932—BUT total PP3/PM1 not exceeding STRONG
	PP3_+3	REVEL score <0.931 & >0.879—BUT total PP3/PM1 not exceeding STRONG
	PP3_moderate	REVEL score <0.878 & >0.773—BUT total PP3/PM1 not exceeding STRONG
	PP3_supporting	REVEL score <0.772 & >0.644
	BP4_supporting	REVEL score <0.29 & >0.184
	BP4_moderate	REVEL score <0.183 & >0.053—BUT total PP3/PM1 not exceeding STRONG
	PP3_-3	REVEL score <0.052 & >0.017—BUT total PP3/PM1 not exceeding STRONG
	BP4_strong	REVEL score ≤0.016—BUT total PP3/PM1 not exceeding STRONG

**Table 2:** 2020 ACMG/AMP Bayesian scoring system published by Tavtigian *et al*. and modified in this study for the stringent classification of likely benign variants

A.		
Evidence Strength	Point scale	
	Pathogenic	Benign
Intermediate	0	0
Supporting	1	−1
Moderate	2	−2
Strong	4	−4
Very strong	8	−8

B.		
Classification	Points range (Tavtigian 2020)	Points range (this study)
Pathogenic	≥10	≥10
Likely Pathogenic	6 to 9	6 to 9
Uncertain	0 to 5	−4 to 5
Likely Benign^a^	−1 to −6	−5 to −6
Benign	≤−7	≤−7

**A.** An evidence strength-dependent allocation of points towards variant reclassification. **B.** The sum of points for all evidence applicable are used to determine the pathogenicity of a variant.

^a^In this study, the Bayesian classification points range for classification of likely benign was conservatively modified to match the 2015 ACMG/AMP guidelines by corresponding to 1 strong benign and 1 supporting benign criteria.

### *SCN5A* variant Brugada syndrome penetrance estimates

To study the effect of various data types (functional data, hotspot location, *in silico* predictions, and ACMG classifications) on stratifying variant penetrance, we employed a recently described Bayesian binomial strategy penetrance approach.^31^ This approach estimates the penetrance of variants discovered as secondary findings,^11^ a key mode of large, contemporary sequencing studies.^31^ Here, we first approximate variant penetrance using a BrS prevalence obtained from the literature (1:2000 based on a meta-analysis of 26 studies spanning 388 237 individuals).^32^ Variant penetrance was then calculated by comparing variants’ allele counts in a previously published BrS cohort (Walsh *et al*.^15^) vs a population database (gnomAD^22^). To test how different evidence types stratified penetrance, we summed the above values across variants within certain bins. These penetrance estimates and confidence intervals were calculated using the *penetrance* R function obtained from https://github.com/ImperialCardioGenetics/variantfx/tree/main/PenetrancePaper.^31^ Specifically, we predicted *SCN5A*-BrS_penetrance as:


(4) BrS_penetrance=(BrS_prevalence)*(AF_case)/(AF_population)

where AF_case refers to allele counts obtained from the BrS cohort, AF_population refers to allele count from the population database gnomAD v4.1, and BrS_prevalence is the prevalence of BrS. Hypothetically, this could also be applied to other large biobank studies such as All of Us or UK BioBank.

We implemented this approach to calculate penetrance estimates for bins of variants across ranges of several predictor scores/classifications: AlphaMissense score (using calibrated score ranges),^12^ REVEL score (using calibrated score ranges),^12^ hotspot criteria (PM1_mod, PM1_supp, or none, as described above), pCD *Z*-score (in bins of width 1), ACMG/AMP functional evidence criterion (PS3/BS3 evidence levels), and ACMG/AMP classification. For each bin, we determined total BrS cases,^15^ gnomAD allele count, average AF for cases, and average AF of gnomAD variants.^22^

The above model explicitly incorporates epidemiological data and relevant differences in AC ascertainment to account for penetrance within different study applications. Notably, the case definition for this referral cohort was focused on electrocardiogram (EKG) changes with sodium channel blocker provocation rather than spontaneous EKG patterns.^15^

### Derivation of odds ratios

Odds ratios (OR) of BrS diagnosis for subgroups of variants (based on functional data, structural location, *in silico* predictions, and ACMG/AMP classifications) were calculated using Eq. 5.


(5) $$\mathrm{Odds}\;\mathrm{ratio}=\;\frac{a}{b}{/}_{\frac{c}{d}}$$

where *a* is the count of BrS cases in each subgroup of variant heterozygotes, b is the count of BrS cases without the variants, *c* is the count of gnomAD with variants in each subgroup, *d* is the count of gnomAD without variants. Control counts were taken from gnomAD v4.1 and total BrS cases from the BrS cohort.^15^

### Statistical analyses

To stabilize variance and approximate normality of measurements, we evaluated potential power transformations using the Box–Cox procedure of WT cells from each research location (see Supplementary data online, *methods* for more details). Based on the transformed WT dataset, we calculated the minimum number of cells required to detect a 25% difference in pCD at 90% power with 95% confidence intervals was 36 cells (see Supplementary data online, *methods* for more details^14^). Shapiro-Wilk normality tests were used to determine the distribution of the benign variants. Comparison of the APC dataset to existing manual patch-clamp data was performed using a Spearman correlation.

## Results

### A calibrated APC assay to enhance *SCN5A* variant classification

In this study, we report the function of each variant relative to that of *SCN5A* WT control cells included in every APC experiment (*Figure 1*). A total of 4615 WT cells were measured, the largest reported collection to date. Data was transformed to approximate a normal distribution using a square-root transformation, which fell within the confidence intervals of the Box–Cox procedure (*λ*: 0.49, 95% CI = 0.39–0.59 for VCCRI and *λ*: 0.45, 95% CI = 0.37–0.52 for VUMC; see Supplementary data online, *methods* for more details). To establish a normal functional range that accounts for the biological variation of benign *SCN5A* variants present in the general population, 25 *SCN5A* benign variant controls^14^ were re-examined with data collected at both sites and aggregated. The mean normalized values of pCD for these benign controls were utilized to determine the SD required for establishing the *Z*-scores for *SCN5A*-BrS variants (see Supplementary data online, *Table S5*). Importantly, the mean normalized values of pCD for these benign controls passed the normality test (see Supplementary data online, *Figure S1*), which allows us to use this range to define a *Z*-score for each variant (see Methods section for details). As the disease-causing mechanism of *SCN5A*-BrS mainly arises from a loss of Na_V_1.5 function, the threshold for distinguishing normal and abnormal *SCN5A* variants was set at 2 SD below the population mean of our 25 benign controls (i.e. a *Z*-score of ≤ −2 is considered abnormal). The assay achieved a 96% sensitivity (95% CI = 79–99), and 96% specificity (95% CI = 80–99). Based on ClinGen's formula for calculating Odds of Pathogenicity (OddsPath),^13^ the updated *SCN5A*-BrS assay had OddsPath scores of 23.96 and 0.043 for pathogenic and benign evidence, respectively, which is equivalent to PS3_strong and BS3_strong evidence in the ACMG/AMP guidelines (see Supplementary data online, *Figure S2*). However, we conservatively downgraded BS3_strong to BS3_moderate to account for potential functional mechanisms that were not captured by this assay when we assessed the function of VUS (see discussion). Addition of gating parameters altered the evidence for only 3 variants (p.Cys683Gly, p.Arg1308Pro, p.Arg1631His) but did not alter the classification we could apply based on separation of control variants.^14^ Therefore, for subsequent analyses, we only used pCD. However, for completeness, all pCD and gating data are shown in Supplementary data online, *Tables S5*–*6*.

### A diverse range of functional effects for *SCN5A* variants in the Brugada syndrome cohort

*SCN5A* variants can have a spectrum of effects on channel function.^17^ To improve the understanding of the molecular pathogenesis of BrS related to *SCN5A* dysfunction, we employed our calibrated APC assay to quantify the function of the 252 missense and in-frame indel *SCN5A* variants reported in the BrS cohort (*Figure 1* and Supplementary data online, *Table S6*).^14^ We first measured Na_V_1.5 sodium pCD at −30 mV, from a holding potential of −90 mV, the primary molecular correlate of *SCN5A*-BrS. Example traces are shown in *Figure 1A*, and pCD measurements for all variants are presented in Supplementary data online, *Table S6*. Reduced pCD was observed in 146 variants (58%). This includes 100 variants (40%) with severe LOF, 25 (10%) with moderate LOF and 21 (8%) with mild LOF, corresponding to PS3_strong, PS3_moderate, and PS3_supporting evidence, respectively. Conversely, 106 variants (42%) had pCD values within the normal range (76 with BS3_moderate and 30 with BS3_supporting evidence, *Figure 1C*). Where there is previous data available for manual patch-clamp analysis of these variants, there is close agreement between our APC results and the published manual recordings (Spearman r = 0.75, 95% CI = 0.61–0.84, *n* = 90; Supplementary data online, *Figure S3*).^33^ The chief outlier is p.Ile1659Val, which was previously reported as LOF,^34^ yet displayed normal or slight gain-of-function in both our labs.

Six missense variants in the cohort had aggregate SpliceAI scores above 0.5 and 20 missense variants had scores between 0.2 and 0.5 (see Supplementary data online, *Table S3*). Of these 26 variants, 10 had current densities in the normal range (*Z* > −2) and thus are candidates to disrupt Na_V_1.5 function primarily through a splicing mechanism,^21^ which is not captured by this APC assay.

### *SCN5A* variant function differs across protein domains

**Figure 2:**
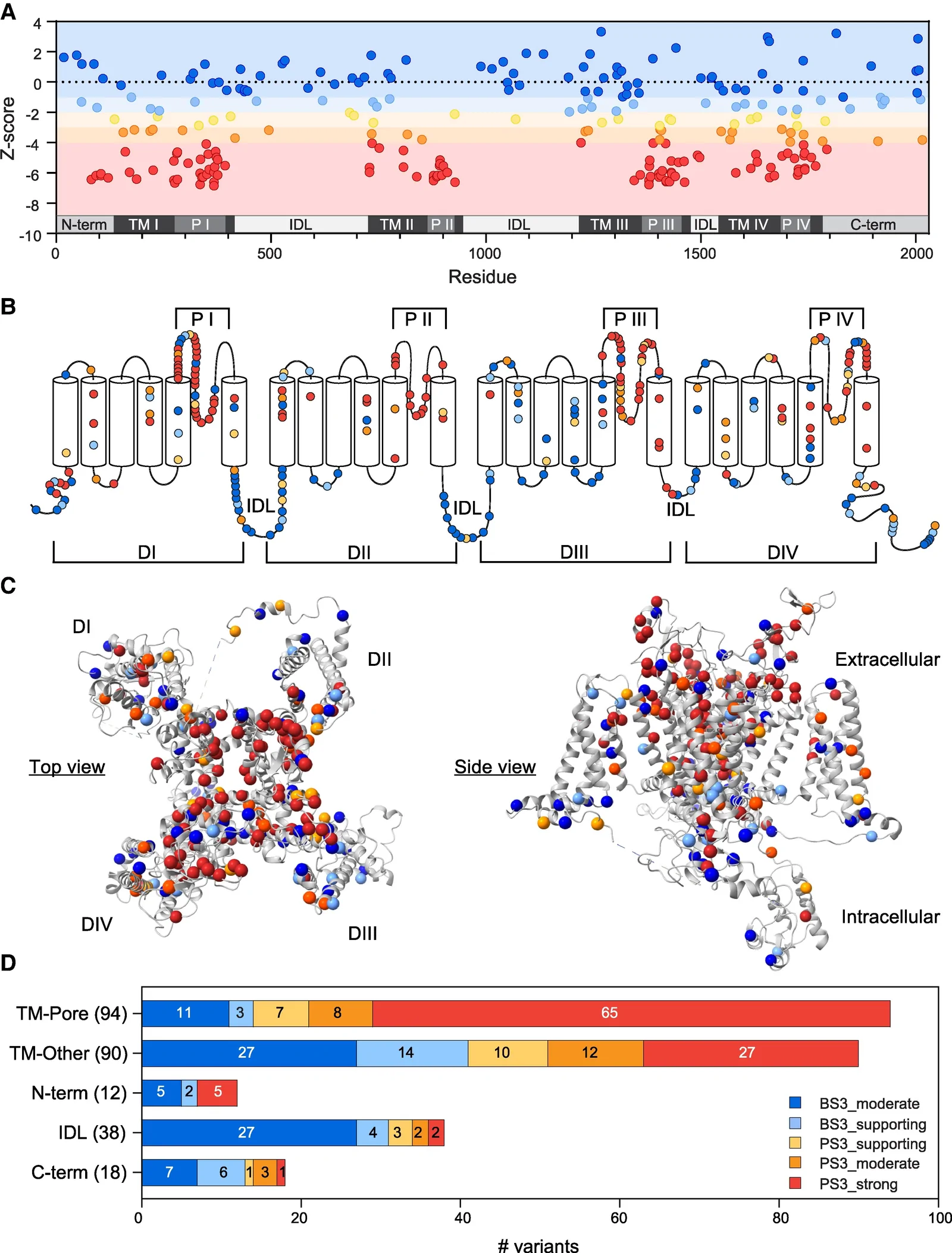
Functional scores of *SCN5A*-BrS variants by protein domain. *(A*) *Z*-scores of variants in the cohort plotted against position in the protein. *(B*) Two-dimensional topological view of Na_V_1.5. *(C*) Three-dimensional structure of hNa_V_1.5 (PDB: 8VYJ^24^). Many N-term, IDL, and C-term variants are not present in the structure and are omitted from this plot. *(D*) Counts of functional criteria by location in protein. *A*–*D*. Variants are colour-coded by ACMG/AMP classification level as defined in the legend in the lower right. Most variants found in the BrS cases are in the transmembrane and pore loop regions, and these regions are also enriched for loss-of-function variants. N-term indicates N-terminus; TM, transmembrane; P, pore; IDL, interdomain linker; C-term, C-terminus; pCD, peak current density

Previous studies have shown that the most severe LOF variants tend to be located in the transmembrane pore domains of ion channels.^17^ Our data are consistent with this general trend. Most missense and in-frame indel variants in this cohort were in transmembrane regions (*Figure 2A–C*; N = 184/252, 73.0%, see methods for definition of different regions of the channel). The transmembrane pore loop regions showed the highest fraction of LOF variants: 80/94 (85%) variants had LOF scores (*Z* ≤ −2), whilst in the transmembrane domains outside the pore loop region, only 49/90 (54%) variants were LOF. Despite the enrichment of LOF variants in the transmembrane regions, there were nonetheless 55 rare and ultra-rare variants with normal function in these hotspot domains (*Figure 2D*), for example, p.Ala364Val in the DI pore loop (BS3_moderate, *Z* = −0.06; present in a single BrS case in the BrS cohort and a single participant in gnomAD v4.1) which also is predicted to have normal splicing by SpliceAI (see Supplementary data online, *Table S3*). Variants within the IDL and C-terminus had the highest fractions of functionally normal variants; 31/38 (82%) and 13/18 (72%), respectively (*Figure 2D*).

### Loss-of-function variants are more likely to be ultra-rare

**Figure 3:**
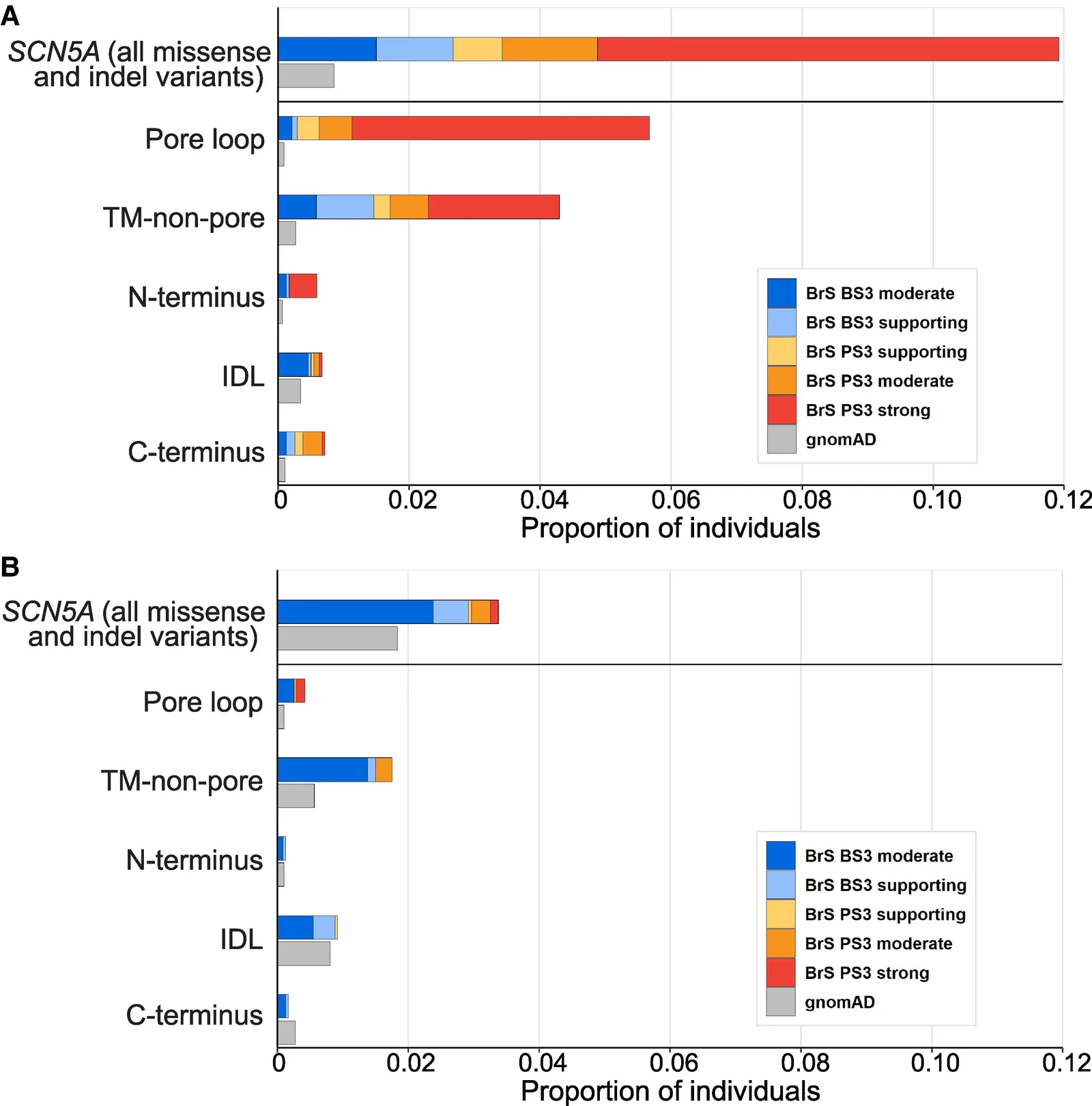
Proportion of individuals that have ultra-rare or rare *SCN5A* variants by protein domains. *(A*) Ultra-rare variants (gnomAD v4.1 FAF <0.00001) are located mostly in the transmembrane (TM) domains and are functionally abnormal except for the few in the Inter-domain Linker (IDL) region. *(B*) Rare variants with a gnomAD v4.1 FAF between 0.00001 and 0.001 are mostly functionally normal in all domains

Genetic evidence suggests that the impact of *SCN5A* missense variants on BrS depends on both where it lies within the Na_V_1.5 protein and how rare the variant is in the population.^15,35^ Case–control studies have consistently noted a strong enrichment of ultra-rare (gnomAD FAF <0.00001) missense variants in *SCN5A* in BrS cohorts compared to control or population datasets.^15,35^ We therefore investigated whether both rare and ultra-rare variants in this BrS cohort are enriched in structured protein domains (*Figure 3A*). Overall, the proportion of BrS cases with LOF variants (PS3_supporting to strong rule applied) falls comfortably within the case excess for the overall analysis as well as for the five regions separately, indicating that the assay is not generally overestimating reduced sodium channel function or variant pathogenicity. Most LOF variants fall into the ultra-rare category. For BrS patients with an ultra-rare *SCN5A* variant, 77.6% of the time, the variant had LOF. There was only a marginal excess of rare (0.001 >gnomAD v4.1 FAF >0.00001) *SCN5A* missense variants observed in BrS cases. Only 13.6% of rare variants had LOF, and these are entirely restricted to the transmembrane region (for these European-ancestry cases) (*Figure 3B*). Overall, 9.25% of patients in the entire BrS cohort had an ultra-rare LOF (PS3 supporting-strong) variant, and 0.46% had a rare LOF variant.

### Integration of functional data improves *SCN5A* variant classification

**Figure 4:**
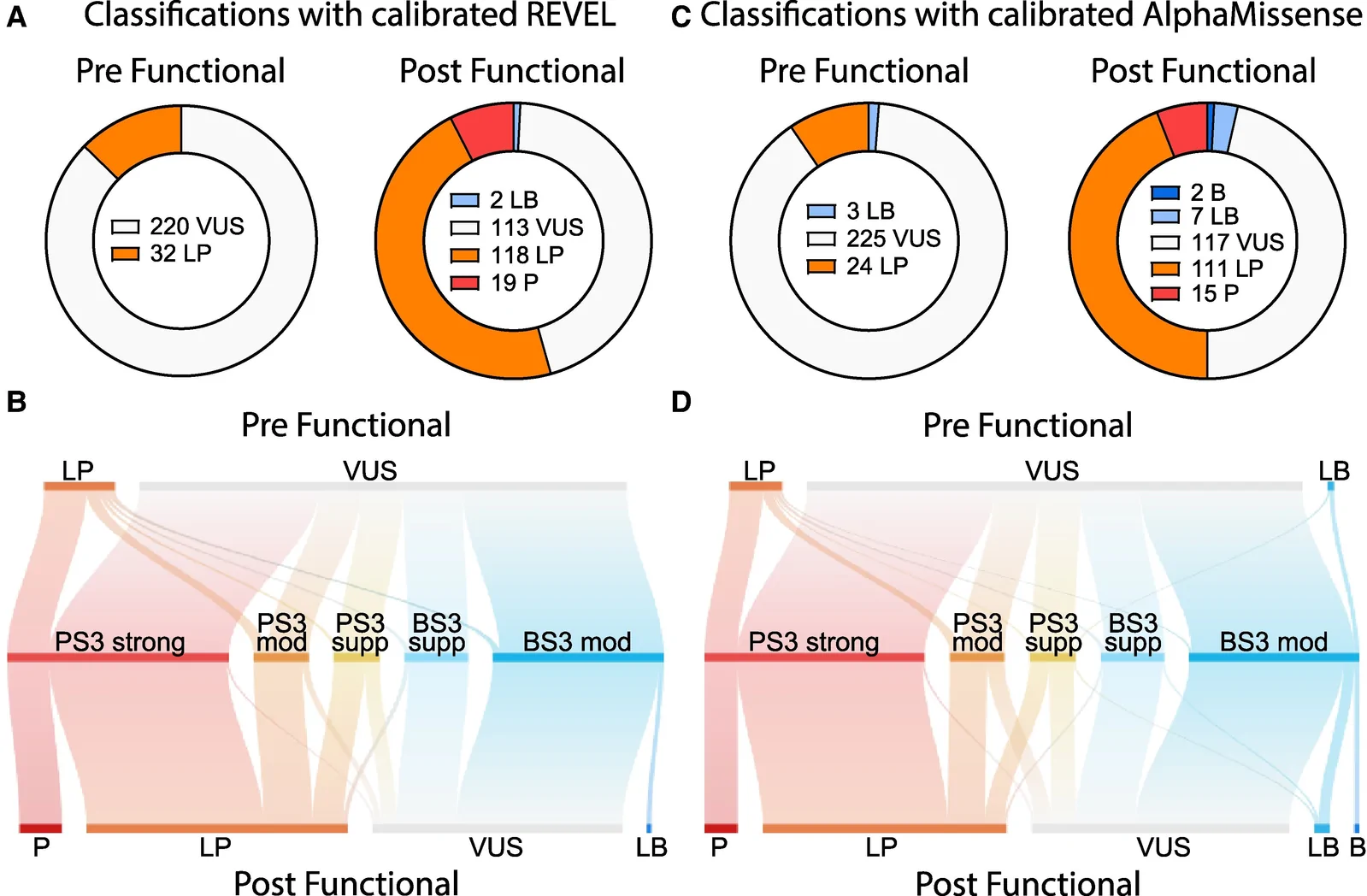
Impact of functional data (PS3/BS3) on variant classifications. Variant classifications for 252 *SCN5A* variants from the BrS cohort were pre- and post-applying functional APC evidence (PS3_strong to BS3_moderate). Variants were classified using population frequency (BS1), variant rarity (PM2_supporting), hotspot evidence (PM1 at a maximum of moderate), in-frame indel (PM4), and case enrichment (PS4). A through D, Calibrated *in silico* predictions (PP3/BP4) from REVEL (*A*, *B*) or AlphaMissense (*C*, *D*) were applied, at a maximum level of 4 points when combined with PM1. Sankey plots show flow between functional evidence and variant classifications for calibrated REVEL (*B*) and calibrated AlphaMissense (*D*). A full list of criteria is provided in *Table 1*

We next evaluated the impact of the 252-variant APC functional dataset on variant classifications. To do this, we combined the functional data with hotspot, AF, case counts, and computational predictors (see Supplementary data online, *Table S7*). Two computational tools, REVEL^27^ and AlphaMissense^28^ were used. We assessed variant classifications for REVEL and AlphaMissense using the ClinGen-recommended calibration cut-offs and classification guidelines for these *in silico* tools.^12,29^ Using calibrated REVEL alone, 220 variants were classified as VUS and 32 as LP. REVEL plus APC data led to 2 LB, 113 VUS, 118 LP, and 19 P classifications (*Figure 4A* and *B*, Supplementary data online, *Table S8*). For AlphaMissense, baseline classifications were 3 LB, 225 VUS, and 24 LP. Adding APC data revised these to 2 B, 7 LB, 117 VUS, 111 LP, and 15 P (*Figure 4C* and *D*, Supplementary data online, *Table S8*). These findings demonstrate that incorporating functional data enables significant variant reclassification, with many VUS shifting to LP and LP to P, across various classification rules.

### *SCN5A* variant function is the best predictor of penetrance in the Brugada syndrome cohort

**Figure 5:**
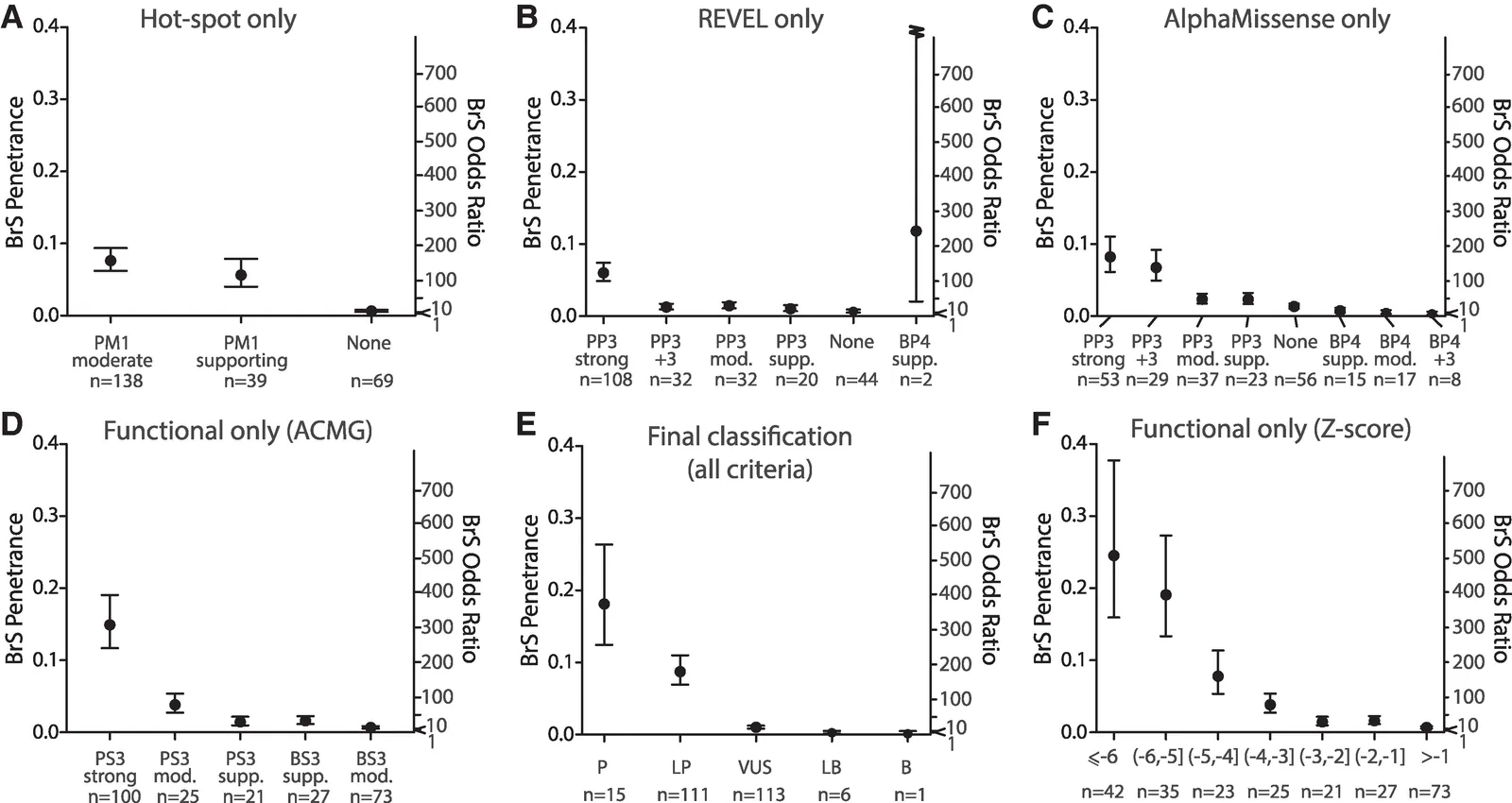
BrS Penetrance and odds ratio based on classifications or variant features. Variants were stratified by hotspot evidence (*A*), calibrated REVEL (*B*), calibrated AlphaMissense (*C*), functional evidence (using ACMG/AMP evidence categories) (*D*), final classification (integrating all criteria) (*E*) or functional evidence derived from pCD *Z*-score with brackets inclusive and parentheses exclusive of respective values (*F*). Data shown represent mean and 95% CI (see Supplementary data online, *Table S9*)

Like many Mendelian disease genes, *SCN5A* variants linked to BrS exhibit incomplete penetrance.^8^ We therefore investigated whether our high-throughput functional data could improve stratification of variant-specific penetrance. We first assessed aggregate BrS penetrance, OR, and risk ratio for BrS by variant classification. The complete set of penetrance, OR, and risk ratio values are presented in *Figure 5* and Supplementary data online, *Table S9*. Hotspot evidence (when used alone) had only a moderate predictive ability, with a maximum penetrance of 0.08 (95% CI = 0.062–0.093) and OR of 165 (95% CI = 141–194) for BrS for PM1_moderate (*Figure 5A*). Calibrated REVEL and AlphaMissense when used alone likewise had only a moderate ability to stratify BrS risk, with a maximum penetrance of 0.06 (95% CI = 0.05–0.07) and OR of 129 (95% CI = 110–151) for REVEL PP3_strong and 0.08 (95% CI = 0.06–0.11) and OR of 169 (95% CI = 130–220) for AlphaMissense PP3_strong (*Figure 5B* and *C*). Functional data had the strongest predictive ability of any single criterion. When binned by ACMG evidence level, functional data had a maximum penetrance of 0.15 (95% CI = 0.12–0.19) and OR of 318 (95% CI = 259–391) for PS3_strong (*Figure 5D*). For the final ACMG/AMP classifications (using hotspot, calibrated AlphaMissense and functional data), P variants had a penetrance of 0.18 (95% CI = 0.12–0.26) and OR of 371 (95% CI = 261–528), LP variants had a penetrance of 0.09 (95% CI = 0.07–0.11) and OR of 185 (95% CI = 153–223), and VUS, LB, and B variants each had a penetrance below 0.01 (*Figure 5E*). When we used a more granular analysis of the functional data, binned by *Z*-score, there was an even higher predictive ability, with a maximum penetrance of 0.25 (95% CI = 0.16–0.38) and OR of 501 (95% CI = 332–757) for *Z* ≤ −6 (*Figure 5F*).

## Discussion

*SCN5A* LOF variants are a major cause of BrS.^36,37^ However, many *SCN5A* missense variants are classified as VUS due to limited available evidence. In this study, we demonstrated the utility of functional data, obtained using a validated high-throughput APC assay^14^ for improving the classification of variants identified from a large cohort of BrS patients.^15^ Of 252 tested variants, 146 had LOF, including 100 with strong LOF. Incorporating this functional evidence with other classification criteria resulted in the reclassification of 48% of VUS when using ACMG/AMP guidelines and current ClinGen recommendations.^10,12,13,29^ Furthermore, the degree of functional perturbation significantly correlated with BrS OR in a clinical cohort and penetrance for a secondary finding population.

Our dataset is the most extensive collection of *SCN5A* variant functional data collected to date. For those variants where there are data already available, from previous manual patch-clamp studies,^33^ there was strong concordance between our APC data and the previous data (see Supplementary data online, *Figure S3*). A previous study of BrS patients found that those with patch-clamp-confirmed LOF *SCN5A* variants had a higher incidence of life-threatening arrhythmic events than those with normal-function *SCN5A* variants.^36^ This finding is consistent with our OR and penetrance estimates. Similarly, our finding that LOF variants were enriched in transmembrane domains, especially the pore loop region, is consistent with previous studies showing that individuals harbouring transmembrane domain variants experience worse outcomes.^37^ We also note that LOF variants were much more likely to be ultra-rare (gnomAD FAF <0.00001), i.e. 78% of ultra-rare variants showed LOF, whereas only 13.6% of rare variants (FAF between 0.00001 and 0.001) had LOF, with all the LOF rare variants located in the transmembrane domains.

Variant classification remains a major challenge for the clinical genetics community. Furthermore, the concordance between diagnostic laboratories can be as low as 34%.^38,39^ To try to overcome the inconsistencies, the ACMG/AMP has introduced a unified framework, for integrating evidence from population data, segregation data, computational data, and functional data.^10^ In the BrS cohort studied here, using the latest recommendations for incorporation of hotspot and computational tools^12,27^ resulted in classification of the 252 variants into 32 P/LP and 220 VUS when applying REVEL or 24 P/LP, 225 VUS, and 3 LB/B when applying AlphaMissense. The addition of our APC functional data enabled the reclassification of nearly half of all VUS to LP when combined with calibrated *in silico* predictions using REVEL (107 reclassified) or AlphaMissense (108 reclassified).^12,27^ Our functional data also prevented functionally normal VUS from being reclassified to LP due to strong-level pathogenic predictions from REVEL (14 variants) or AlphaMissense (7 variants). Finally, our normal functional evidence also helped reclassify 2 and 6 rare VUS to LB when calibrated REVEL and AlphaMissense were used, respectively. Overall, functional data enabled reclassification of 48.6% of VUS when using calibrated REVEL and 48.0% of VUS when using calibrated AlphaMissense. As variant classifications continue to evolve, including ongoing efforts by ClinGen Variant Curation Expert Panels to standardize gene-specific criteria, we anticipate that the current work will provide ‘real-world’ results to improve genetic evaluation of inheritable heart disease.^12,13,29^

Despite the overall success of the APC functional data in improving variant reclassification, we note that in rare instances, there are discordant results between *in vitro* and *in vivo* systems. For example, the variant p.Asp1274Asn, which we report as normal function (*Z* = 0.28) confirms previous *in vitro* studies in HEK293 cells but is different from the reduced current density observed in mouse models, which also show a dilated cardiomyopathy phenotype that recapitulates the clinical phenotype.^40^ The mechanism of this difference remains unclear. Our *in vitro* APC system also cannot identify variants that alter splicing. Amongst the 252 variants studied here, 26 had SpliceAI scores suggesting they could disrupt splicing, and 10 out of those 26 had normal function in the APC assay. Thus, it is possible that our assay has missed a LOF phenotype in some of these variants. These exceptions do not outweigh the overall strength of the current approach, especially since our assay has been carefully calibrated on a large set of control P/LP and B/LB variants. Nevertheless, to prevent a misleadingly ‘reassuring’ false-negative functional result from influencing care, we suggest limiting normal-function evidence to a maximum of moderate-level evidence.^26^

The incomplete penetrance of LOF variants in ‘Mendelian’ disease genes has been increasingly recognized, especially with the availability of relatively unselected population and biobank cohorts.^22,31,41^ For BrS, only about 20% of BrS patients have rare *SCN5A* variants, and these variants furthermore typically exhibit incomplete penetrance.^42^ There are also numerous examples of *SCN5A* variants causing variable phenotypes, including no phenotype, within a single family.^43–46^ In addition to penetrance within family studies and ascertained clinical workup, genotype-first precision medicine will increasingly rely on adjudication of variants observed in unascertained populations, such as those involved as secondary findings.^11^ Previous work on *SCN5A* variant penetrance has focused on penetrance estimates incorporating proactive integration of variant features using an expectation-maximization framework integrating several study designs.^42,47^ Here, we used an approach established for cardiomyopathy by McGurk *et al*. to use binned sets of variant features or variant classifications to estimate penetrance in an unascertained population.^31^ Although *in silico* predictions correlated with BrS penetrance, the severity of LOF in the APC assay was the feature that best correlated with risk for BrS. These data suggest that when APC data are available, they could be used to inform the value of return of secondary findings, an increasingly important clinical challenge,^11^ and especially for patients with normal EKGs. Our analysis specifically focuses on *SCN5A*-BrS penetrance, which only partially captures the genetic architecture of BrS. We note that manifestation of BrS is likely modulated by many features not modelled in this study, including demographics, environmental influences, polygenic pathways,^9,48,49^ and/or yet-to-be-identified monogenic influences.^50^

### Limitations

Although *SCN5A* is a pleiotropic gene and *SCN5A* variants can cause a variety of arrhythmia and heart failure phenotypes (including Long QT Type 3, Dilated Cardiomyopathy, and Multiple Ectopic Premature Purkinje Contractions), our functional assay is currently calibrated only for the *SCN5A*-BrS relationship. The frequency of *SCN5A* LOF variants is reported to differ among distinct genetic ancestry groups,^51^ and this analysis includes few cases of non-European and non-East Asian ancestry. While patient demographics and common genetic background influence BrS risk, we could not account for these variables within the available data. Our assay used a cDNA form of *SCN5A* that cannot model variant effects on splicing. Variants found in BrS patients with moderate or high SpliceAI scores can be further studied with complementary splicing assays.^21,52^ Our experiments were performed in HEK293 cells and not cardiomyocytes. While ion channel biophysics are similar across cellular models, there are rare examples of variants with discordant functional properties between HEK293 cells and cardiomyocytes.^40^ Additionally, Na_V_1.5 undergoes extensive post-translational phosphorylation within intracellular regions.^53^ It is possible that cardiomyocyte-specific phosphorylation events are not captured in our HEK293 assay. We note, however, that none of the variants in this study were among any of the known 42 phosphorylation residues. We also do not model dominant-negative effects in this study. Future functional assays in other model systems and contexts may further refine our understanding of the disease impact of *SCN5A* variants. Finally, penetrance estimates are limited by varied allele frequencies in referral populations and phenotype definitions (EKG findings as criteria in the BrS cohort).

BrS is a rare disease with a population prevalence of approximately 1 in 2000. Our data suggest that the presence of a LOF variant in *SCN5A* increases this risk to approximately 1 in 5. This provides the mathematical basis for the high odds ratios observed, which can reach values as high as 500. We recognize that both the penetrance and odds ratio estimates are influenced by multiple sources of uncertainty, including variability in the population prevalence of BrS and limitations related to small sample sizes in certain calculations. Therefore, we recommend interpreting the presented penetrance and odds ratio values as approximate estimates rather than precise figures. Though our penetrance estimates were best correlated to disease risk, the exact prediction of the penetrance of BrS is affected by confounding factors, including the variability of the EKG of family members and medication use. Conversely, it is possible that BrS patients are included in the gnomAD population. Despite these uncertainties, the data demonstrate a clear trend of increasing disease risk with more severe impairments in channel function.

## Conclusions

In this study, we deployed a calibrated functional assay on 252 rare missense and in-frame indel variants detected in a large BrS cohort. Integration of these data with allele frequency, case-enrichment, hotspot, and computational evidence enabled the reclassification of approximately 48% of VUS according to the latest ACMG/AMP criteria. We also demonstrated a strong correlation between the extent of LOF and variant penetrance. Our dataset and approach should further aid in the clinical management of individuals with *SCN5A* variants and their families undergoing BrS evaluation. In the future, other *SCN5A* variants implicated in gain-of-function channelopathies such as LQTS Type 3 may also be amenable to APC-based functional characterization. Our approach could also be adapted to other genes implicated in channelopathy disorders.^54^ While the overall experimental framework would be similar, assay protocols may need to be tailored and validated using gene- and disease-specific B/LB and P/LP control variants.

## Supplementary Material

ehaf874_Supplementary_Data

## Supplementary data

### Data Availability

All experimental and *in silico* data generated and used in this analysis are included in this study. Code to analyse these data is available at the Glazer Lab GitHub site: https://github.com/GlazerLab/SCN5A_SP_BrS_cohort and at the VCCRI GitHub site: https://github.com/VCCRI/SCN5A_SyncroPatch. Variant functional data and classifications will be submitted to ClinVar upon acceptance of the manuscript. All included patient data were derived from previously published, deidentified data.

## References

[1] Cerrone M, Costa S, Delmar M. The genetics of Brugada syndrome. Annu Rev Genomics Hum Genet 2022;23:255–74. 10.1146/annurev-genom-112921-011200 35567276

[2] Milman A, Andorin A, Gourraud JB, Postema PG, Sacher F, Mabo P, et al Profile of patients with Brugada syndrome presenting with their first documented arrhythmic event: data from the Survey on Arrhythmic Events in BRUgada Syndrome (SABRUS). Heart Rhythm 2018;15:716–24. 10.1016/j.hrthm.2018.01.014 29325976

[3] Brugada P, Brugada J. Right bundle branch block, persistent ST segment elevation and sudden cardiac death: a distinct clinical and electrocardiographic syndrome. A multicenter report. J Am Coll Cardiol 1992;20:1391–6. 10.1016/0735-1097(92)90253-j 1309182

[4] Dereci A, Yap SC, Schinkel AFL. Meta-analysis of clinical outcome after implantable cardioverter-defibrillator implantation in patients with Brugada syndrome. JACC Clin Electrophysiol 2019;5:141–8. 10.1016/j.jacep.2018.09.005 30784682

[5] Hosseini SM, Kim R, Udupa S, Costain G, Jobling R, Liston E, et al Reappraisal of reported genes for sudden arrhythmic death: evidence-based evaluation of gene validity for Brugada syndrome. Circulation 2018;138:1195–205. 10.1161/circulationaha.118.035070 29959160 PMC6147087

[6] Wilde AAM, Amin AS. Clinical spectrum of SCN5A mutations: long QT syndrome, Brugada syndrome, and cardiomyopathy. JACC Clin Electrophysiol 2018;4:569–79. 10.1016/j.jacep.2018.03.006 29798782

[7] Mizusawa Y, Wilde AA. Brugada syndrome. Circ Arrhythm Electrophysiol 2012;5:606–16. 10.1161/circep.111.964577 22715240

[8] Wijeyeratne YD, Tanck MW, Mizusawa Y, Batchvarov V, Barc J, Crotti L, et al SCN5A mutation type and a genetic risk score associate variably with Brugada syndrome phenotype in SCN5A families. Circ Genom Precis Med 2020;13:e002911. 10.1161/circgen.120.002911 33164571 PMC7748043

[9] Barc J, Tadros R, Glinge C, Chiang DY, Jouni M, Simonet F, et al Genome-wide association analyses identify new Brugada syndrome risk loci and highlight a new mechanism of sodium channel regulation in disease susceptibility. Nat Genet 2022;54:232–9. 10.1038/s41588-021-01007-6 35210625 PMC9376964

[10] Richards S, Aziz N, Bale S, Bick D, Das S, Gastier-Foster J, et al Standards and guidelines for the interpretation of sequence variants: a joint consensus recommendation of the American College of Medical Genetics and Genomics and the Association for Molecular Pathology. Genet Med 2015;17:405–24. 10.1038/gim.2015.30 25741868 PMC4544753

[11] Miller DT, Lee K, Abul-Husn NS, Amendola LM, Brothers K, Chung WK, et al ACMG SF v3.2 list for reporting of secondary findings in clinical exome and genome sequencing: a policy statement of the American College of Medical Genetics and Genomics (ACMG). Genet Med 2023;25:100866. 10.1016/j.gim.2023.100866 37347242 PMC10524344

[12] Bergquist T, Stenton SL, Nadeau EAW, Byrne AB, Greenblatt MS, Harrison SM, et al Calibration of additional computational tools expands ClinGen recommendation options for variant classification with PP3/BP4 criteria. Genet Med 2025;27:101402. 10.1016/j.gim.2025.101402 40084623 PMC12208618

[13] Brnich SE, Abou Tayoun AN, Couch FJ, Cutting GR, Greenblatt MS, Heinen CD, et al Recommendations for application of the functional evidence PS3/BS3 criterion using the ACMG/AMP sequence variant interpretation framework. Genome Med 2020;12:3. 10.1186/s13073-019-0690-2 PMC6938631 31892348

[14] Ma JG, O’Neill MJ, Richardson E, Thomson KL, Ingles J, Muhammad A, et al Multisite validation of a functional assay to adjudicate SCN5A Brugada syndrome-associated variants. Circ Genom Precis Med 2024;17:e004569. 10.1161/circgen.124.004569 38953211 PMC11335442

[15] Walsh R, Lahrouchi N, Tadros R, Kyndt F, Glinge C, Postema PG, et al Enhancing rare variant interpretation in inherited arrhythmias through quantitative analysis of consortium disease cohorts and population controls. Genet Med 2021;23:47–58. 10.1038/s41436-020-00946-5 32893267 PMC7790744

[16] Morales J, Pujar S, Loveland JE, Astashyn A, Bennett R, Berry A, et al A joint NCBI and EMBL-EBI transcript set for clinical genomics and research. Nature 2022;604:310–5. 10.1038/s41586-022-04558-8 35388217 PMC9007741

[17] Glazer AM, Wada Y, Li B, Muhammad A, Kalash OR, O’Neill MJ, et al High-throughput reclassification of SCN5A variants. Am J Hum Genet 2020;107:111–23. 10.1016/j.ajhg.2020.05.015 32533946 PMC7332654

[18] Ng CA, Farr J, Young P, Windley MJ, Perry MD, Hill AP, et al Heterozygous KCNH2 variant phenotyping using Flp-In HEK293 and high-throughput automated patch clamp electrophysiology. Biol Methods Protoc 2021;6:bpab003. 10.1093/biomethods/bpab003 33884304 PMC8046900

[19] Jiang C, Richardson E, Farr J, Hill AP, Ullah R, Kroncke BM, et al A calibrated functional patch-clamp assay to enhance clinical variant interpretation in KCNH2-related long QT syndrome. Am J Hum Genet 2022;109:1199–207. 10.1016/j.ajhg.2022.05.002 35688147 PMC9300752

[20] Jaganathan K, Kyriazopoulou Panagiotopoulou S, McRae JF, Darbandi SF, Knowles D, Li YI, et al Predicting splicing from primary sequence with deep learning. Cell 2019;176:535–48.e524. 10.1016/j.cell.2018.12.015 30661751

[21] O’Neill MJ, Yang T, Laudeman J, Calandranis ME, Harvey ML, Solus JF, et al ParSE-seq: a calibrated multiplexed assay to facilitate the clinical classification of putative splice-altering variants. Nat Commun 2024;15:8320. 10.1038/s41467-024-52474-4 39333091 PMC11437130

[22] Karczewski KJ, Francioli LC, Tiao G, Cummings BB, Alföldi J, Wang Q, et al The mutational constraint spectrum quantified from variation in 141,456 humans. Nature 2020;581:434–43. 10.1038/s41586-020-2308-7 32461654 PMC7334197

[23] Li Z, Jin X, Wu T, Zhao X, Wang W, Lei J, et al Structure of human Na(v)1.5 reveals the fast inactivation-related segments as a mutational hotspot for the long QT syndrome. Proc Natl Acad Sci U S A 2021;118:e2100069118. 10.1073/pnas.2100069118 33712541 PMC7980460

[24] Biswas R, López-Serrano AL, Purohit A, Ramirez-Navarro A, Huang HL, Grandinetti G, et al Structural basis of human Na(v)1.5 gating mechanisms. Proc Natl Acad Sci U S A 2025;122:e2416181122. 10.1073/pnas.2416181122 40366698 PMC12107192

[25] Lomize AL, Todd SC, Pogozheva ID. Spatial arrangement of proteins in planar and curved membranes by PPM 3.0. Protein Sci 2022;31:209–20. 10.1002/pro.4219 34716622 PMC8740824

[26] Thomson KL, Jiang C, Richardson E, Westphal DS, Burkard T, Wolf CM, et al Clinical interpretation of KCNH2 variants using a robust PS3/BS3 functional patch-clamp assay. HGG Adv 2024;5:100270. 10.1016/j.xhgg.2024.100270 38219013 PMC10840334

[27] Ioannidis NM, Rothstein JH, Pejaver V, Middha S, McDonnell SK, Baheti S, et al REVEL: an ensemble method for predicting the pathogenicity of rare missense variants. Am J Hum Genet 2016;99:877–85. 10.1016/j.ajhg.2016.08.016 27666373 PMC5065685

[28] Cheng J, Novati G, Pan J, Bycroft C, Žemgulytė A, Applebaum T, et al Accurate proteome-wide missense variant effect prediction with AlphaMissense. Science 2023;381:eadg7492. 10.1126/science.adg7492 37733863

[29] Pejaver V, Byrne AB, Feng BJ, Pagel KA, Mooney SD, Karchin R, et al Calibration of computational tools for missense variant pathogenicity classification and ClinGen recommendations for PP3/BP4 criteria. Am J Hum Genet 2022;109:2163–77. 10.1016/j.ajhg.2022.10.013 36413997 PMC9748256

[30] Tavtigian SV, Harrison SM, Boucher KM, Biesecker LG. Fitting a naturally scaled point system to the ACMG/AMP variant classification guidelines. Hum Mutat 2020;41:1734–7. 10.1002/humu.24088 32720330 PMC8011844

[31] McGurk KA, Zhang X, Theotokis P, Thomson K, Harper A, Buchan RJ, et al The penetrance of rare variants in cardiomyopathy-associated genes: a cross-sectional approach to estimating penetrance for secondary findings. Am J Hum Genet 2023;110:1482–95. 10.1016/j.ajhg.2023.08.003 37652022 PMC10502871

[32] Vutthikraivit W, Rattanawong P, Putthapiban P, Sukhumthammarat W, Vathesatogkit P, Ngarmukos T, et al Worldwide prevalence of Brugada syndrome: a systematic review and meta-analysis. Acta Cardiol Sin 2018;34:267–77. 10.6515/ACS.201805_34(3).20180302B 29844648 PMC5968343

[33] Kroncke BM, Glazer AM, Smith DK, Blume JD, Roden DM. SCN5A (NaV1.5) variant functional perturbation and clinical presentation: variants of a certain significance. Circ Genom Precis Med 2018;11:e002095. 10.1161/CIRCGEN.118.002095 29728395 PMC5941942

[34] Cordeiro JM, Barajas-Martinez H, Hong K, Burashnikov E, Pfeiffer R, Orsino AM, et al Compound heterozygous mutations P336L and I1660V in the human cardiac sodium channel associated with the Brugada syndrome. Circulation 2006;114:2026–33. 10.1161/circulationaha.106.627489 17075016 PMC1989773

[35] Kapplinger JD, Tester DJ, Alders M, Benito B, Berthet M, Brugada J, et al An international compendium of mutations in the SCN5A-encoded cardiac sodium channel in patients referred for Brugada syndrome genetic testing. Heart Rhythm 2010;7:33–46. 10.1016/j.hrthm.2009.09.069 20129283 PMC2822446

[36] Ishikawa T, Kimoto H, Mishima H, Yamagata K, Ogata S, Aizawa Y, et al Functionally validated SCN5A variants allow interpretation of pathogenicity and prediction of lethal events in Brugada syndrome. Eur Heart J 2021;42:2854–63. 10.1093/eurheartj/ehab254 34219138

[37] Yamagata K, Horie M, Aiba T, Ogawa S, Aizawa Y, Ohe T, et al Genotype-phenotype correlation of *SCN5A* mutation for the clinical and electrocardiographic characteristics of probands with Brugada syndrome. Circulation 2017;135:2255–70. 10.1161/CIRCULATIONAHA.117.027983 28341781

[38] Harrison SM, Dolinsky JS, Knight Johnson AE, Pesaran T, Azzariti DR, Bale S, et al Clinical laboratories collaborate to resolve differences in variant interpretations submitted to ClinVar. Genet Med 2017;19:1096–104. 10.1038/gim.2017.14 28301460 PMC5600649

[39] Amendola LM, Muenzen K, Biesecker LG, Bowling KM, Cooper GM, Dorschner MO, et al Variant classification concordance using the ACMG-AMP variant interpretation guidelines across nine genomic implementation research studies. Am J Hum Genet 2020;107:932–41. 10.1016/j.ajhg.2020.09.011 33108757 PMC7675005

[40] Watanabe H, Yang T, Stroud DM, Lowe JS, Harris L, Atack TC, et al Striking in vivo phenotype of a disease-associated human SCN5A mutation producing minimal changes in vitro. Circulation 2011;124:1001–11. 10.1161/circulationaha.110.987248 21824921 PMC3297976

[41] Forrest IS, Chaudhary K, Vy HMT, Petrazzini BO, Bafna S, Jordan DM, et al Population-based penetrance of deleterious clinical variants. JAMA 2022;327:350–9. 10.1001/jama.2021.23686 35076666 PMC8790667

[42] Kroncke BM, Smith DK, Zuo Y, Glazer AM, Roden DM, Blume JD. A Bayesian method to estimate variant-induced disease penetrance. PLoS Genet 2020;16:e1008862. 10.1371/journal.pgen.1008862 32569262 PMC7347235

[43] Smits JP, Koopmann TT, Wilders R, Veldkamp MW, Opthof T, Bhuiyan ZA, et al A mutation in the human cardiac sodium channel (E161K) contributes to sick sinus syndrome, conduction disease and Brugada syndrome in two families. J Mol Cell Cardiol 2005;38:969–81. 10.1016/j.yjmcc.2005.02.024 15910881

[44] Rossenbacker T, Carroll SJ, Liu H, Kuipéri C, de Ravel TJ, Devriendt K, et al Novel pore mutation in SCN5A manifests as a spectrum of phenotypes ranging from atrial flutter, conduction disease, and Brugada syndrome to sudden cardiac death. Heart Rhythm 2004;1:610–5. 10.1016/j.hrthm.2004.07.001 15851228

[45] Grant AO, Carboni MP, Neplioueva V, Starmer CF, Memmi M, Napolitano C, et al Long QT syndrome, Brugada syndrome, and conduction system disease are linked to a single sodium channel mutation. J Clin Invest 2002;110:1201–9. 10.1172/jci15570 12393856 PMC150793

[46] Bezzina C, Veldkamp MW, van Den Berg MP, Postma AV, Rook MB, Viersma JW, et al A single Na(+) channel mutation causing both long-QT and Brugada syndromes. Circ Res 1999;85:1206–13. 10.1161/01.res.85.12.1206 10590249

[47] O’Neill MJ, Sala L, Denjoy I, Wada Y, Kozek K, Crotti L, et al Continuous Bayesian variant interpretation accounts for incomplete penetrance among Mendelian cardiac channelopathies. Genet Med 2022;25:100355. 10.1016/j.gim.2022.12.002 36496179 PMC9992222

[48] Cerrone M, Remme CA, Tadros R, Bezzina CR, Delmar M. Beyond the one gene–one disease paradigm. Circulation 2019;140:595–610. 10.1161/CIRCULATIONAHA.118.035954 31403841 PMC6697136

[49] Ishikawa T, Masuda T, Hachiya T, Dina C, Simonet F, Nagata Y, et al Brugada syndrome in Japan and Europe: a genome-wide association study reveals shared genetic architecture and new risk loci. Eur Heart J 2024;45:2320–32. 10.1093/eurheartj/ehae251 38747976

[50] Bersell KR, Yang T, Mosley JD, Glazer AM, Hale AT, Kryshtal DO, et al Transcriptional dysregulation underlies both monogenic arrhythmia syndrome and common modifiers of cardiac repolarization. Circulation 2023;147:824–40. 10.1161/CIRCULATIONAHA.122.062193 36524479 PMC9992308

[51] Walsh R, Mauleekoonphairoj J, Mengarelli I, Bosada FM, Verkerk AO, van Duijvenboden K, et al A rare noncoding enhancer variant in SCN5A contributes to the high prevalence of Brugada syndrome in Thailand. Circulation 2024;151:31–44. 10.1161/circulationaha.124.069041 39391988 PMC11670919

[52] O’Neill MJ, Wada Y, Hall LD, Mitchell DW, Glazer AM, Roden DM. Functional assays reclassify suspected splice-altering variants of uncertain significance in Mendelian channelopathies. Circ Genom Precis Med 2022;15:e003782. 10.1161/CIRCGEN.122.003782 36197721 PMC9772980

[53] Lorenzini M, Burel S, Lesage A, Wagner E, Charrière C, Chevillard PM, et al Proteomic and functional mapping of cardiac NaV1.5 channel phosphorylation sites. J Gen Physiol 2021;153:e202012646. 10.1085/jgp.202012646 33410863 PMC7797897

[54] Kim JB . Channelopathies. Korean J Pediatr 2014;57:1–18. 10.3345/kjp.2014.57.1.1 24578711 PMC3935107

