# Supplementary material for: Automated patch clamp data improve variant classification and penetrance stratification for SCN5A–Brugada syndrome
Source: Eur Heart J. 2025 Nov 18;47(33):4679–93. doi: 10.1093/eurheartj/ehaf874 (PMC12678899; doi:10.1093/eurheartj/ehaf874)
Supplement: Supplementary file 1 [file ehaf874_supplementary_data.zip › Supplement.docx]

**Supplemental Data**

Cohort-scale automated patch clamp data improves variant classification and penetrance stratification for *SCN5A*-Brugada Syndrome

*Cohort-Scale SCN5A Variant Functional Data*

Matthew J. O’Neill, MD, PhD^1,2†^, Joanne G. Ma, BSc^3,4,†^, Jessa L. Aldridge, PhD^5^, Joseph F. Solus, PhD^5^, Genevieve R. Harvey^5^, Paige H. Roberson^5^, Julien Barc, PhD^6,7^, Connie R. Bezzina, PhD^7,8^, Dan M. Roden, MD^5,9^, Roddy Walsh, PhD^8,10^, Jamie I. Vandenberg, MBBS, PhD^3,4,^*, Andrew M. Glazer, PhD^5,^*, Chai-Ann Ng, PhD^3,4,^*

^1^Department of Medicine, Brigham and Women’s Hospital, Boston, MA, USA

^2^Clinical Fellow in Medicine, Harvard Medical School, Boston, MA, USA

^3^Mark Cowley Lidwill Research Program in Cardiac Electrophysiology, Victor Chang Cardiac Research Institute, Darlinghurst, NSW, Australia

^4^School of Clinical Medicine, St Vincent's Healthcare Clinical Campus, Faculty of Medicine and Health, UNSW Sydney, Australia

^5^Vanderbilt Center for Arrhythmia Research and Therapeutics (VanCART), Division of Clinical Pharmacology, Department of Medicine, Vanderbilt University Medical Center, Nashville, TN, USA

^6^Nantes Université, CNRS, INSERM, L'institut du thorax, Nantes, France

^7^European Reference Network for Rare, Low Prevalence and Complex Diseases of the Heart: ERN GUARD-Heart, The Netherlands

^8^Department of Experimental Cardiology, Amsterdam Cardiovascular Sciences, University of Amsterdam, Amsterdam UMC, Amsterdam, The Netherlands

^9^Departments of Pharmacology, and Biomedical Informatics, Vanderbilt University Medical Center, Nashville, TN, USA

^10^Cardiovascular and Genomics Research Institute, City St. George’s University of London, London, UK

^†^These authors contributed equally

*Co-corresponding authors

**Table of Contents**

[Supplemental Methods	3](#_Toc208303864)

[Supplemental Figures	5](#_Toc208303865)

[Figure S1: Test of normality show a gaussian distribution of benign variant controls.	5](#_Toc208303866)

[Figure S2: Multisite OddsPath Recalibration.	6](#_Toc208303867)

[Figure S3: Relationship between manual and automated patch clamp results for *SCN5A* variants.	7](#_Toc208303868)

[Figure S4: Odds Ratio estimates based on the classifications or variant features.	8](#_Toc208303869)

# Supplemental Methods

**BoxCox transformation**

For each location (VCCRI and VUMC), data were modeled using a constant-only linear model, and the Box–Cox transformation parameter (λ) was estimated with 95% confidence intervals. Analyses were performed in R using the car::powerTransform function. For the VCCRI dataset, the estimated λ was 0.49 (95% CI = 0.39–0.59), while for the VUMC dataset, the estimated λ was 0.45 (95% CI = 0.37–0.52). In both cases, the 95% confidence intervals excluded λ = 0 (log transformation) and λ = 1 (no transformation) but included λ = 0.5, supporting the square root transformation as an appropriate method for stabilizing variance and improving normality. All subsequent analyses were performed on square root–transformed values.

**Sample size calculation**

Sample size calculations were performed to determine the number of cells required to detect a 25% and a 50% difference at 90% power with 95% confidence intervals. The sample size power equation used:

$$k= \frac{n^{2}}{n^{1}}=1$$

$$n_{1}= \frac{{\left( \sigma_{1}^{2}+\frac{\sigma_{2}^{2}}{K} \right)\left( z_{1-\frac{\alpha}{2}}+z_{1-\beta} \right)}^{2}}{\Delta^{2}}$$

To detect 25% difference at 90% power, where α = 0.05

$$n_{1}= \frac{{\left( {32.7}^{2}+\frac{{32.7}^{2}}{1} \right)\left( 1.96+1.28 \right)}^{2}}{{25}^{2}}=36$$

$$n_{2}=K*n_{1}=36$$

Where, Δ = |μ_2_-μ_1_| = absolute difference between two means, σ_1_, σ_2_ = variance of mean #1 and #2, n_1_ = sample size for group #1, n_2_ = sample size for group #2, α = probability of type I error (usually 0.05), β = probability of type II error (usually 0.2), z = critical Z value for a given α or β, and k = ratio of sample size for group #2 to group #1.

We found that a minimum of 36 cells was required for each variant to detect a 25% difference at 90% power.

**Sensitivity and specificity calculation**

$$sensitivity = \frac{TP}{TP+FN}$$

$$specificity = \frac{TN}{TN+FP}$$

Where, TP = true positive, FN = false negative, TN = true negative and FP = false positive.

$$CI = \frac{\left( p̂+\frac{z^{2}}{2n} \right)\pm\sqrt[z]{\frac{\hat{p}(1-\hat{p})}{n}+\frac{z^{2}}{{4n}^{2}}}}{1+\frac{z^{2}}{n}}$$

Where, *CI* = confidence interval, *p̂* = proportion (sensitivity or specificity that is being calculated), *n* = number of cases (TP+FN or TN+FP), *z* = 1.96 for 95% CI.

**Risk ratio calculations**

Risk ratios (RR) of BrS diagnosis for subgroups of variants (based on functional data, structural location, *in silico* predictions, and ACMG/AMP classifications) were calculated using equation X.

$Risk ratio=\frac{\frac{a}{(a+c)}}{\frac{b}{(b+d)}}$	(X)

where a, b, c, and d are defined as in equation 5.

# Supplemental Figures

|  | pCD |
| --- | --- |
| Test for normal distribution |  |
| Shapiro-Wilk test |  |
| W | 0.9267 |
| P value | 0.0731 |
| Passed normality test (alpha=0.05)? | Yes |
| P value summary | ns |
| Number of values | 25 |

## **Figure S1: Test of normality show a gaussian distribution of benign variant controls.**

Peak current densities for the 25 benign variant controls, weighted across Vanderbilt University Medical Center and Victor Chang Cardiac Research Institute, were found to be normally distributed. Shapiro-Wilk test p = 0.0731.

## **Figure S2: Multisite OddsPath Recalibration.**

(A) Confusion matrix of showing the sensitivity (96%, 95% CI = 79–99) and specificity (96%, 95% CI = 80–99) of the assay. (B) OddsPath scores post recalibration of *SCN5A*-BrS physiological voltage assay in accordance with ClinGen SVI Working Group guidelines. This assay can provide evidence at strong levels for both BS3 and PS3, benign and pathogenic, but will be conservatively applied at BS3_moderate in this assay.

## **Figure S3: Relationship between manual and automated patch clamp results for *SCN5A* variants.**

A literature search for manual patch clamp data performed in HEK293 cells found current densities for 90/252 variants studied in this cohort. Comparison of manual patch clamp data and our automated patch clamp data revealed Spearman r = 0.75 (95% CI = 0.61–0.84). I1659V, previously reported to have LOF, showed normal function at both our independent sites where automated patch clamping was performed.

## **Figure S4: Odds Ratio estimates based on the classifications or variant features.**

Variants were stratified by hot-spot evidence (A), calibrated AlphaMissense (B), calibrated REVEL (C), functional evidence (using ACMG/AMP evidence categories) (D), final classification (integrating all criteria) (E) or functional evidence derived from pCD Z-score with brackets inclusive and parenthesis exclusive of respective values (F). Data shown represents mean and 95% CI (Table S9).
